# The women’s health needs study among women from countries with high prevalence of female genital mutilation living in the United States: Design, methods, and participant characteristics

**DOI:** 10.1371/journal.pone.0302820

**Published:** 2024-05-31

**Authors:** Ghenet Besera, Margaret Christine Snead, Mary Goodwin, Ashley Smoots, Connie L. Bish, Alicia Ruiz, Ayeesha Sayyad, Sabrina Avripas, Petry Ubri, Roy Ahn, Vicki Pineau, Nicole Warren, Doris Mukangu, Crista E. Johnson-Agbakwu, Howard Goldberg, Ekwutosi Okoroh, on behalf of The WHNS Study Team

**Affiliations:** 1 Epidemic Intelligence Service, Centers for Disease Control and Prevention, Atlanta, Georgia, United States of America; 2 Division of Violence Prevention, Centers for Disease Control and Prevention, Atlanta, Georgia, United States of America; 3 Division of Reproductive Health, Centers for Disease Control and Prevention, Atlanta, Georgia, United States of America; 4 Independent Consultant, Atlanta, Georgia, United States of America; 5 Division of Birth Defects and Infant Disorders, Centers for Disease Control and Prevention, Atlanta, Georgia, United States of America; 6 NORC at the University of Chicago, Chicago, Illinois, United States of America; 7 Johns Hopkins University School of Nursing, Washington, DC, United States of America; 8 Amani Women Center, Atlanta, Georgia, United States of America; 9 Collaborative in Health Equity, Office of Health Equity, The University of Massachusetts, Worcester, Massachusetts, United States of America; 10 Obstetrics & Gynecology, UMass Memorial Health, Worcester, Massachusetts, United States of America; 11 Division of Preventive and Behavioral Medicine, Population and Quantitative Health Sciences, University of Massachusetts T.H. Chan Medical School, Worcester, Massachusetts, United States of America; National Defence University - Kenya, KENYA

## Abstract

**Background:**

The Women’s Health Needs Study (WHNS) collected information on the health characteristics, needs, and experiences, including female genital mutilation (FGM) experiences, attitudes, and beliefs, of women aged 18 to 49 years who were born, or whose mothers were born, in a country where FGM is prevalent living in the US. The purpose of this paper is to describe the WHNS design, methods, strengths and limitations, as well as select demographic and health-related characteristics of participants.

**Methods:**

We conducted a cross-sectional survey from November 2020 –June 2021 in four US metropolitan areas, using a hybrid venue-based sampling (VBS) and respondent-driven sampling (RDS) approach to identify women for recruitment.

**Results:**

Of 1,132 participants, 395 were recruited via VBS and 737 RDS. Most were born, or their mothers were born, in either a West African country (Burkina Faso, Guinea, Mali, Mauritania, Sierra Leone, The Gambia) (39.0%) or Ethiopia (30.7%). More than a third were aged 30–39 years (37.5%) with a majority who immigrated at ages ≥13 years (86.6%) and had lived in the United States for ≥5 years (68.9%). Medicaid was the top health insurer (52.5%), followed by private health insurance (30.5%); 17% of participants had no insurance. Nearly half of women reported 1–2 healthcare visits within the past 12 months (47.7%). One in seven did not get needed health care due to cost (14.8%). Over half have ever used contraception (52.1%) to delay or avoid pregnancy and 76.9% had their last pelvic and/or Papanicolaou (pap) exam within the past 3 years. More than half experienced FGM (55.0%). Nearly all women believed that FGM should be stopped (92.0%).

**Conclusion:**

The VBS/RDS approach enabled recruitment of a diverse study population. WHNS advances research related to the health characteristics, needs, and experiences of women living in the US from countries where FGM is prevalent.

## Introduction

Female Genital Mutilation (FGM), defined as “all procedures involving partial or total removal of the external female genitalia or other injury to the female genital organs for non-medical reasons,” [1] can result in a range of acute and chronic adverse health outcomes [1–4]. The continuance of FGM is largely driven by social and cultural beliefs that vary across communities; these beliefs include ensuring marriageability, modesty, and premarital virginity [5, 6]. While FGM is primarily practiced in countries throughout Africa, Asia, and the Middle East, it also occurs in other countries/continents, including when women migrate from geographic areas where FGM is commonly practiced [7]. Although the exact number of women and girls with FGM living in the United States is unknown, previous studies have estimated the number of potentially affected women and girls who were at risk for undergoing FGM [8, 9]. For example, Goldberg et al. [8] applied country-specific prevalence rates from the Demographic and Health Surveys (DHS) [10] and the Multiple Indicator Cluster Surveys [11] to estimate the number of women at risk of or potentially affected by FGM among women and girls, from practicing countries, living in the United States. Goldberg et al. found that approximately 513,000 women and girls in the United States may have experienced or were at risk for FGM [8]. These indirect estimates did not collect information directly from women or account for other factors that can potentially affect FGM practices, such as age of immigration, acculturation, or changes in attitudes after migration [8].

Primary data collected directly from women living in the US from countries where FGM is prevalent is limited. A scoping review [12] found that many US-based research studies tend to focus on a single country of origin or metropolitan community within the United States; additionally, they tend to have relatively small study population sizes (<100 participants). Lastly, there is limited information about the physical or emotional health needs of women of reproductive age (18 to 49) who have immigrated from or whose mothers are from high-prevalence FGM countries. Even less information is available on their reproductive health, such as contraceptive use/need, childbearing history, and access to/use of reproductive preventive health care services. Additional research is needed to better understand and address the health needs of this population in the US.

The Women’s Health Needs Study (WHNS) was designed to gather information on health needs and experiences, including the experience of FGM, from US women aged 18 to 49 who were born, or whose mothers were born, in a country where FGM is prevalent. The overall study objectives were to describe participant’s 1) access to and use of preventive health services; 2) reproductive and mental health experiences; and 3) FGM experiences, attitudes, and beliefs. The purpose of this paper is to describe the WHNS design, methods, strengths and limitations as well as select demographic and health-related characteristics of participants.

## Methods

The Centers for Disease Control and Prevention (CDC)’s Division of Reproductive Health (DRH) contracted with NORC at the University of Chicago, and with subject matter expertise consultation from a panel of experts and community members, designed and implemented WHNS, a cross-sectional, multi-site survey.

### Study eligibility

Women were eligible for WHNS if they were: 1) aged 18 to 49 years; 2) born or their mother was born in Burkina Faso, Egypt, Eritrea, Ethiopia, Guinea, Mali, Mauritania, Sierra Leone, Somalia, Sudan, or The Gambia (hereafter called study “countries of origin”), and 3) able to complete a survey interview in one of the languages for which a translated questionnaire and interviewer were available (i.e., Amharic, Arabic, English, French, Oromo, Somali, or Tigrinya). The study countries of origin were included in eligibility criteria for WHNS because their estimated national FGM prevalence was at least 65 percent in the most recent nationally representative survey that collects demographic and health data (e.g., Demographic and Health Survey [10], or Multiple Indicator Cluster Surveys [11]) and population concentrations within the metropolitan area of study sites.

### Study design

We employed a hybrid venue-based sampling (VBS) [13] and respondent-driven sampling (RDS) [14] approach to identify and screen potentially eligible women. These methods are often used when surveying hard-to-reach or hidden populations [13, 15–17] or similarly in types of situations where the survey’s content is sensitive and may require more specialized culturally appropriate recruitment efforts [18–20]. VBS is a time-space sampling method used to recruit respondents at venues and at times where or when the study population is expected to gather [13]. RDS is a form of peer-based study recruitment that incorporates features designed to limit and adjust for biases in traditional snowball sampling [14]. For WHNS RDS sampling, each VBS participant who completed an interview was given a unique code word and invited to recruit up to three eligible women (the RDS participants) from her social network to potentially participate in the study. The code word was used to maintain confidentiality and track RDS referrals.

### Study instruments, translations, and interviewers

A 6-question screener was administered to assess study eligibility (S1 File). The WHNS questionnaire (S2 File) included 62 questions, some of which were adapted from surveys such as the National Survey of Family Growth [21], and the Pregnancy Risk Assessment Monitoring System [22]. Questions about FGM experience and type were developed using examples from the DHS [10] and previous FGM studies conducted in the United States and Europe [19, 20, 23, 24].

Translations of study materials were conducted by certified translation vendors. Moreover, translated study materials were reviewed by at least two community WHNS staff for accuracy and to ensure cross cultural equivalency was attained across the various languages.

WHNS interviewers were women who spoke English and at least one other study language (for which they passed a required standard language proficiency assessment). They were also familiar with or from the WHNS study communities and the study’s selected countries of origin. All WHNS staff were trained by NORC with a standard training manual and interactive presentations that covered human subjects research ethics, the WHNS protocol, and study implementation procedures. WHNS staff also received specialized training on the topic of FGM by an obstetrician-gynecologist with subject matter expertise.

### Study implementation

We conducted a pilot study from September to November 2019 in one US metropolitan community to test the methods and procedures for the WHNS, including the acceptability and feasibility of collecting sensitive health information from the study population. For the pilot study, we obtained 101 face-to-face survey interviews, demonstrated that the study was acceptable to participants, and demonstrated that the VBS/RDS sampling methodology was feasible. Following the pilot study, we made minor modifications to the WHNS human subjects’ protocol, screener, and questionnaire based on analysis of pilot results and feedback from the pilot implementing Community-Based Organizations (CBO), WHNS interviewers, and study participants. Changes, implemented after the pilot study, included: 1) refinements to the content and conversational flow of the screener and main survey questionnaire, 2) replacing open-ended with categorical response options in the questionnaire, and 3) increasing the maximum possible incentive total amounts from the planned $35 to $55 for participation in the study. Main study participants received $25 for their interview and $10 for each additional participant (up to three) recruited from their social network, totaling $55 to cover expenses they may have incurred (e.g., costs for cell phone calls while participating in the interviews, childcare expenses during this time). To maximize confidentiality, all reimbursements were made using a codename.

After completing the pilot study, the full study was conducted from November 2020 to June 2021 in four US metropolitan areas (Atlanta, Minneapolis, New York, and Washington, DC). In each metropolitan site, NORC worked with a CBO to implement the study. These areas were selected because each had large concentrations of residents from the study’s countries of origin [25]. We established study population size goals for the numbers of completed survey interviews by country of origin and metropolitan area, based on the estimated size and composition of the populations in each of the four metropolitan areas. Not all countries with high FGM prevalence had large enough concentrations within the four metropolitan areas. For example, Djibouti has very high FGM prevalence, but population density was low within the metropolitan areas where recruitment took place. The minimum number of completed interviews that would be needed to detect statistically significant differences between women from East and West African countries and between younger and older women was approximately 1,100 women. The West African countries were grouped into a single regional category. This grouping was made because the American Community Survey (ACS) data, which was used to develop the sample size goals and weight the sample, did not separate out the individual West African countries included in our study (except for Sierra Leone) [25].

Country-of-origin sample size goals were 289 participants for West African countries (Burkina Faso, Guinea, Mali, Mauritania, Sierra Leone, The Gambia); 242 Ethiopian participants; 159 Somalia participants; 132 Sudanese participants, 128 Eritrean participants, and 76 Egyptian participants. Study population size goals also varied by Metropolitan area based on their estimated population densities from the study countries-of-origin: 406 in Atlanta, 176 in Minneapolis, 315 in New York, and 254 in Washington, DC.

Consistent with other studies using VBS and RDS methods, the pilot study relied on face-to-face recruitment and interviewing [13, 15, 17]. However, the COVID-19 pandemic mitigation measures implemented in 2020–2021 (e.g., stay-at-home orders, physical distancing) necessitated adaptations. Physical venues were replaced with “virtual” venues, and a secure/restricted WHNS Resource Website was created for participants to access study contact information, consent forms, visual aids, and a list of CBOs and resources (such as healthcare or mental health providers).

### Institutional research approvals & informed consent

Both the CDC and NORC Human Subjects Institutional Review Boards (IRB) reviewed and approved the WHNS Human Subjects Protocol and all amendments to the study design (including the pilot and full study) (CDC IRB Protocol 7123; NORC IRB Protocol 17.02.08 Project 8031/8630). Paperwork Reduction Act approval for data collection was received from the Office of Management and Budget (OMB # 0920–1264). Implementing CBOs relied on NORC’s IRB approval for local data collection activities. Although no personally identifiable information was collected during the study process, the study covered sensitive topics (e.g., reproductive health, health care access, and FGM) and received a Certificate of Confidentiality as well as a National Institute of Justice Privacy Certificate. In efforts to further protect participants’ privacy, WHNS interviewers obtained and documented verbal informed consent from potential participants in her preferred language.

### Analysis

We conducted a descriptive analysis of selected participant characteristics, and present both weighted and unweighted percentages and unweighted *Ns*. We also present unweighted *Ns* for “Don’t know” and “Prefer not to answer” responses, however, in most instances these responses are excluded from percentage calculations. All analyses were performed using SAS 9.4.

### Weighting

Two types of weighting adjustments were applied to the survey data. First, we used information about respondents’ self-reported network size (number of family and friends in the community) to create a mathematical model of the recruitment process and weight the sample to help correct for unequal selection probabilities [26]. This approach was needed because study participants with larger family and friends’ networks had a higher probability of being selected into the sample than those with smaller networks. Second, a post-stratification weighting adjustment was applied to consider differences in age and country of origin distributions between the WHNS sample and the population of women eligible for the study [27]. The 2018 ACS was used for the post-stratification weighting because it is considered a reliable source to reduce potential bias due to noncoverage, nonresponse, or other non-sampling errors [28]. Study estimates and measures of the precision of those estimates were constructed using the final weights that include both the RDS and post-stratification and weighting adjustments.

### Variables

This analysis characterizes the WHNS participants using selected variables from the screener (S1 File) and questionnaire (S2 File). From the screener, we report *age group* (18–24, 25–29, 30–39, 40–49) and *country of origin*. Country of origin is a derived variable based on the respondent’s or mother’s country of birth whichever made the participant eligible for WHNS.

From the study questionnaire, we report the *highest level of education completed* (less than a high school diploma, high school diploma/GED, some college/associate degree, bachelor’s degree or higher); *language spoken most often at home* [(Amharic, Arabic, English, Somali, French, Tigrinya, Oromo, Mande Languages (including Mandingo, Soninke, Bambara and other Mande languages, Other)]; *length of time in the United States* [new arrivals (US immigration <5 years ago), established (US immigration ≥5 years ago), born in the United States]; and *immigration generation*. The immigration generation variable was created based on age at immigration [1.0 generation (born in another country and arrived in the United States at ages ≥13 years), 1.5 generation (born in another country arrived at ages <13 years), 2.0 generation (born in the United States, with at least 1 parent born in another country)]. These immigrant generation classifications have been used in previous research and distinguish immigrants who arrived at older ages (≥13 years) from those who were younger (<13 years) and therefore may be more likely to have adopted characteristics (e.g., culture, language) similar to US-born individuals [29].

We also include current *marital status* (married/living with a partner, previously married, never married/lived with a partner); *age at first marriage/cohabitation* (<18, 18–24, 25–29, 30+, never married/cohabitated); *work outside the home* (yes, no); and *whether a member of any club*, *association*, *or religious* organization for people from family’s home country or ethnic/cultural background (yes, no).

As it relates to health status, health coverage, healthcare-seeking behavior, and healthcare provider experiences, we include: self-described *health status* (excellent, very good, good, fair, poor); *respondent’s current health insurance coverage* (Medicaid, no insurance, private); *number of health care visits in past 12 months* (no visits, 1–2 visits, 3–5 visits, more than 5 visits); *would like to have someone interpret when visiting a healthcare provider* (yes, no); and *during the past 12 months*, *needed health care but didn’t get it because they couldn’t afford it* (yes, no).

For reproductive health characteristics, we report: *ever used a contraceptive method* (yes, no); *age at first sexual intercourse* (<18, 18–24, 25–29, 30+, never had sexual intercourse); *timing of the last pelvic exam and/or pap* (≤3 years ago, ≥4 years ago, never); and among women who ever had sexual intercourse, *number of children born alive* (0, 1–2; 3–4, 5+).

As it relates to variables on FGM experience, we included: *comes from a family that practiced FGM* (yes, no, don’t know); *FGM experience* (experienced FGM, did not experience FGM). Among women who experienced FGM, we used a hierarchical categorization of *self-reported FGM type* in the following order: sewn closed (infibulated) (yes, no), flesh removed (yes, no), nicked/no flesh removed (yes, no), do not know type, and prefer not to answer. We also included the respondent’s attitude about *continuation of FGM* (it should be stopped, it should continue as is, depends on the family, mixed feelings).

## Results

Of the 2,110 potentially eligible women, 1,386 contacts were successfully made and 1,272 were screened for the study. Among those who were screened, 1,154 were eligible to participate in the study and 1,132 were interviewed (Table 1). There were 395 women recruited via VBS and 737 by RDS (1,132). Table 1 shows the number of completed WHNS survey interviews by metropolitan area and interview language. Atlanta (n = 389) and New York (n = 315) had the most completed interviews. The three most frequent interview languages were English (n = 374), Amharic (n = 199), and French (n = 154). The individual survey interview lasted an average of 37 minutes.

**Table 1:** Number of completed survey interviews by metropolitan area and language of interviews: Women’s Health Needs Study (WHNS), 2020–21 (N = 1,132).

Characteristic	Number of interviews (n)
**Total**	**1,132**
**Metropolitan Area**	
Atlanta	389
New York	315
Washington D.C.	252
Minneapolis	176
**Interview Language**	
Amharic	199
Arabic	115
English	374
Somali	115
French	154
Tigrinya	92
Oromo	71
Other^a^	12

^a^ Other includes Mande language (e.g., Mandingo, Soninke, Bambara, and other Mande languages)

### Participant characteristics

Unweighted and weighted estimates of selected characteristics of women who participated in WHNS are shown in Table 2. Among WHNS participants, 39.0% were born in or their mothers were born in a West African country and 30.7% in Ethiopia. The West African category was comprised of women from Burkina Faso (n = 137), Guinea (n = 62), Mali (n = 57), Mauritania (n = <10), Sierra Leone (n = 16), and The Gambia (n = 65). More than one third of women were aged 30–39 years (37.5%) and more than half either had some college/associate degree (32.5%) or a bachelor’s degree or higher (29.7%). The languages spoken most often at home were Amharic (21.0%), French (15.3%), Arabic (12.4%), and English (12.3%). The majority (86.6%) of participants migrated to the United States at ages ≥13 years (1.0 generation) and had lived in the United States for at least 5 years (68.9%). Most women (60.7%) were married or living with a partner (24.9%), and more than a third (35.5%) were between the ages of 18 and 24 years at first marriage or cohabitation. More than half of the participants did not work outside the home (53.9%). Most women were not members of any club, association, or religious organization for people from their family’s home country or ethnic/cultural background (62.2%) (Table 2).

**Table 2:** Select demographic and health-related characteristics of the Women’s Health Needs Study (WHNS) Population, 2020–21 (N = 1,132).

Characteristic	Unweighted (%)	Weighted(%)	N^b^
**Total**	**100.0**	**100.0**	**1,132**
**Country of Origin** ^c^			
Egypt	5.2	11.1	59
Eritrea	12.1	2.7	137
Ethiopia	24.3	30.7	275
Somalia	15.3	13.3	173
Sudan	13.0	3.2	147
West African Countries^d^	30.1	39.0	341
**Age (years)**			
18–24	22.8	15.8	258
25–29	12.7	15.6	144
30–39	36.8	37.5	417
40–49	27.7	31.1	313
**Highest level of education completed**			
Less than a high school diploma	24.6	15.1	279
High school diploma/GED	27.0	22.7	306
Some college/Associate degree	28.4	32.5	322
Bachelor’s degree or higher	19.9	29.7	225
**Language spoken most often at home**			
Amharic	16.2	21.0	183
Arabic	15.2	12.4	172
English	15.4	12.3	174
Somali	12.5	11.4	142
French	9.9	15.3	112
Tigrinya	10.7	3.5	121
Oromo	6.6	7.8	75
Mande language^e^	9.8	11.4	111
Other	3.7	4.9	42
**Length of time in the U.S.**			
New arrivals (US immigration < 5 years)	26.2	27.9	295
Established (US immigration ≥ 5 years)	60.6	68.9	684
Born in United States	13.2	3.2	149
Missing/Prefer not to answer^f^	-	-	<10
**Immigration generation**			
1.0 generation (Age of US immigration ≥13 years)	77.8	86.6	876
1.5 generation (Age of US immigration <13 years)	9.0	10.1	101
2.0 generation (Born in United States)	13.2	3.3	149
Unknown/No Response^f^	-	-	<10
**Current marital status**			
Married/living with a partner	55.3	60.7	615
Previously married (widowed, divorced, separated)	14.7	14.4	164
Never married/lived with a partner	29.9	24.9	333
Prefer not to answer^f^	-	-	20
**Age at first marriage/cohabitation (years)**			
<18	7.6	5.6	84
18–24	34.9	35.5	388
25–29	20.1	26.7	224
30+	7.5	8.3	83
Never married/cohabitated	29.9	24.9	333
Prefer not to answer^f^	-	-	20
**Work outside the home**			
Yes	43.3	46.1	490
No	56.7	53.9	641
Prefer not to answer^f^	-	-	<10
**Member of any club, association, or religious organization for people from family’s home country or ethnic/cultural background**			
Yes	34.8	37.8	392
No	65.2	62.2	735
Not Sure/Prefer Not to answer^f^	-	-	<10
**Self-described health status**			
Excellent	28.3	27.0	320
Very good	31.5	30.4	357
Good	25.7	27.6	291
Fair	12.0	12.4	136
Poor	2.5	2.5	28
**Health insurance**			
Private	29.9	30.5	333
Medicaid	52.8	52.5	588
No insurance	17.2	17.0	191
Don’t know^f^	-	-	20
**Gone to clinic or hospital for health care in past 12 months**			
No visits	17.1	15.4	192
1–2 visits	47.7	47.4	536
3–5 visits	20.4	21.4	229
6 or more visits	14.8	15.7	166
Don’t Know/Prefer not to answer^f^	-	-	<10
**Would like to have someone to interpret when visiting a healthcare provider**			
Yes	29.9	26.7	337
No	70.1	73.3	789
Do not have a healthcare provider^f^	-	-	<10
Prefer not to answer^f^	-	-	<10
**During the past 12 months, needed health care but didn’t get it because they couldn’t afford it**			
Yes	15.7	14.8	177
No	84.3	85.2	949
Don’t Know/Prefer not to answer^f^	-	-	<10
**Ever used a contraceptive method**			
Yes	43.2	52.1	483
No	56.8	47.9	634
Don’t Know/Prefer not to answer^f^	-	-	15
**Age at first sexual intercourse**			
<18	14.8	15.5	163
18–24	46.5	50.8	512
25–29	14.9	17.3	164
30+	2.7	2.4	30
Never had sexual intercourse	21.1	14.1	232
Don’t Know/Prefer not to answer^f^	-	-	31
**Timing of last Pelvic Exam and/or Pap**			
≤ 3 years ago	69.8	76.9	777
≥ 4 years ago	7.4	6.0	82
Never	22.8	17.0	254
Don’t Know/Prefer not to answer^f^	-	-	19
**Number of Children Born Alive**			
0	33.9	29.5	383
1–2	33.2	37.3	375
3–4	23.4	25.0	264
5+	9.5	8.1	107
Don’t Know/Prefer not to answer^f^	-	-	<10
**Comes from a family that practiced FGM**			
Yes	68.4	70.4	771
No	29.5	28.3	333
Don’t Know	2.1	1.3	24
Prefer not to answer^f^	-	-	<10
**FGM experience**			
Experienced FGM	52.5	55.0	580
Did not experience FGM	47.5	45.0	524
Don’t Know^f^	-	-	21
Prefer not to answer^f^	-	-	<10
**FGM Type** ^g^			
Sewn closed (infibulation)	33.2	28.9	191
Some flesh removed	52.3	57.4	301
Nicked/No flesh removed	3.1	2.3	18
Don’t know type	11.3	11.4	65
Prefer not to answer^f^	-	-	<10
**Attitudes about continuation of FGM**			
It should be stopped	91.6	92.0	1021
It should continue as is	0.8	0.7	<10
Depends on the family	4.5	4.7	50
Mixed feelings	3.1	2.5	35
Don’t know^f^	-	-	15
Prefer not to answer^f^	-	-	<10

FGM = Female genital mutilation, GED = General Education Development, US = United States

^a^ Accounts for respondent driven sampling method and differences in age and country of origin distributions between the sample and the US population of women, based on results of the 2018 American Community Survey

^b^ Unweighted *N*

^c^ Country of origin is based on participant’s birthplace OR mother’s country of birth if that is what made a woman eligible for study.

^d^ Includes Burkina Faso (n = 137), Guinea (n = 62), Mali (n = 57), Mauritania (n = <10), Sierra Leone (n = 16), and The Gambia (n = 65).

^e^ Mande languages include: Mandingo, Soninke, Bambara, and other Mande languages.

^f^ Excluded from percentage calculation.

^g^ Defined as most severe type of FGM experienced (self-reported) in the following order: sewn closed, some flesh removed, nicked/no flesh removed, don’t know type; excludes 552 who did not experience FGM.

More than half of the women described their health status as either very good (30.4%) or good (27.6%). Most participants reported they were insured, with 52.5% having Medicaid and 30.5% having private health insurance. Nearly half (47.7%) had 1–2 healthcare visits to a clinic or hospital in the past 12 months; 14.8% reported they needed medical care in the past 12 months but did not get it because they could not afford it. More than a quarter of women (26.7%) reported they would like to have an interpreter when visiting a healthcare provider (Table 2).

Related to women’s reproductive health, more than half of the participants reported having ever used a contraceptive method to delay or avoid pregnancy (52.1%) and having, had first sexual intercourse between the ages of 18 and 24 (50.8%); 76.9% had their last pelvic exam and/or pap ≤ 3 years ago. Most women reported having had a live birth, with 37.3% having 1 to 2, 25.0% having 3 to 4, and 8.1% having 5 or more (Table 2).

Regarding FGM, 70.4% of participants reported they came from a family that practiced FGM. More than half of women reported they experienced FGM (55.0%). Among the 580 women who reported experiencing FGM, 57.4% reported they had flesh removed, 28.9% were sewn closed (infibulated), 11.4% did not know the type of FGM they experienced, and 2.3% were nicked or had no flesh removed. The majority reported they believed that FGM should be stopped (92.0%) (Table 2).

## Discussion

We describe the WHNS design, methods, strengths and limitations as well as select demographic and health-related characteristics of women living in the US, aged 18–49 who were born, or whose mothers were born in a country where FGM is prevalent. The WHNS study builds upon previous research on women’s reproductive health and FGM experiences [12]. The hybrid VBS/RDS sampling strategy used enabled the recruitment of a demographically diverse (for age and education) sample of US women from the study’s select countries of origin (S1 Table). Previous literature indicates the utility of such methods when surveying hard-to-reach or hidden populations [13, 15–18].

Community-engaged approaches have been described as beneficial for examining sensitive topics. [12, 30]. Because WHNS addresses sensitive topics, community-engagement was essential to ensuring the information collected would be appropriate and acceptable to participants and their communities. NORC and CDC actively sought input from community members throughout the planning and implementation of this study including during the piloting of WHNS methods and materials. Moreover, the engagement of interviewers familiar with the community and who had the ability to interview women in the languages they preferred may have allowed us to reach some women who might not have otherwise participated, consistent with other research [30, 31]. While a strength of our research was that it was founded in community-engagement, future research may consider using a more robust Community Based Participatory Research approach. This may further improve access to local data, enhance interpretation of findings through the community context, and build community embedded strategies to further enhance community engagement for structural and local interventions [30, 31]. The WHNS community-engaged approach provided a culturally relevant, strategic, and systematic way to design, implement, and then disseminate the study findings.

The WHNS study design (i.e., community-engagement for planning and study implementation, RDS/VBS sampling to improve recruitment) could be expanded to include other metropolitan areas in the US and more countries of origin. WHNS could also be expanded to collect additional information on topics that were not originally included (e.g., violence against women, trauma, chronic health conditions). It may be possible to integrate similar questions into other data collection efforts, such as routine health assessments or community-based surveys. This is consistent with global literature that calls for a more engaged health care sector using a public health and human rights approach, and a multi sectored approach (including community health workers) for FGM prevention efforts [32, 33]. The WHNS design could be adapted to investigate other sensitive topics among populations where a sampling frame is limited or does not exist.

We were also successful in not only meeting our recruitment goals but exceeded them despite concerns that lack of face-to-face interviews and the inclusion of very sensitive questions may cause women to be reluctant to participate in the study. This is likely due to the hybrid VBS/RDS sampling strategy and community engagement employed. Moreover, these methods have been used when surveying hard-to-reach or hidden populations [13, 15–18] or similarly in types of situations where the survey’s content is sensitive and may require more specialized culturally appropriate recruitment efforts [31]. Lastly, only 6 participants began the survey but did not complete it (reasons for not completing the study are unknown), which further illustrates that the study design was successful in collecting data which is consistent with a similar community engaged FGM study [30] as well as findings on other sensitive topics from hard-to-reach populations [31].

### Limitations

WHNS has some limitations. The findings cannot be generalized to similar women in the United States, nor can they be used to estimate the number of US women who may have had, or be at risk for FGM. The WHNS sample only included four US metropolitan areas. Except for Sierra Leone, the ACS data [25] used to establish study sample goals for the number of completed interviews and for weighting did not separate West African countries included in our study. Women from West African countries that were grouped together in our analysis may differ on key characteristics, which we are unable to account for in WHNS results. Therefore, the heterogeneity of women from these West African countries should be taken into account when considering how to address their health care needs. Since the VBS/RDS hybrid sampling approach is not random, there may be coverage error, non-response, and/or self-selection biases that limit the interpretation of findings [14]. For example, the overall results may be more reflective of certain countries of origin or communities that had greater number of study participants versus those with fewer participants.

We were unable to validate self-reported FGM type; self-reported information might potentially introduce recall, response, or social desirability biases regarding FGM status [34–36]. Nearly a third (32%) of participants accessed visual aid images provided for a question about Type 1, 2 or 3 FGM based on the visual aid images (S3 File); however, we did not use this single question (S3 File) for this analysis, and we do not know if the use of visual aid images improved [37] or changed participant’s reporting on FGM types. To minimize these biases, we did not use this single question to asses for FGM type. We instead defined FGM type by using three DHS descriptive questions (S3 File) [10]. These questions asked if flesh had been removed from the genital area, was the genital area nicked without flesh removal, or was the genital area sewn closed. Despite using the three descriptive questions, 12% of participants reported that they did not know their FGM type.

WHNS was designed and implemented during heightened sensitivity about immigration [38] and was completed during the COVID-19 pandemic [39, 40]. The role that these factors may have played in who participated in the WHNS study is unknown. Moreover, we did observe participant’s decreased network sizes and reduced participation in clubs and other social events and activities. It is not possible to estimate the impact these factors may have had on recruitment and participation. Lastly, the WHNS study administration was modified from face-to-face to telephone interviews due to pandemic mitigation efforts, potentially introducing additional coverage error and selection bias. Although the degree of bias is unknown, other researchers have indicated that they successfully modified their study protocol during COVID-19 pandemic [41].

Despite multiple recruitment efforts, WHNS did not successfully reach some of the country-of-origin groups in sufficient numbers. Specifically, women from Egypt and Sierra Leone with a relatively large population presence in the United States [25], were not recruited in sufficient numbers that would allow future country of origin-specific analyses. Additionally, the WHNS sample is more reflective of 1.0 generation (born in another country and arrived in the United States at ages ≥13 years) and 1.5 generation (born in another country arrived at ages <13 years) than 2.0 generation (born in the United States, with at least 1 parent born in another country). This generational breakdown may be due to the study recruitment venues and subsequent social networks included. Therefore, findings are more reflective of foreign-born women than those born in the United States.

Our study population only included women ages 18 to 49 years, so does not include key information about the health, health care needs, and FGM status of younger women of reproductive age (e.g.,15 to 17 years) or women older than 49. Family is referenced within the survey, but it was not defined within the survey, leaving participants to define family based on their own understanding. In efforts to protect privacy, age data was collected as ranges, which further limited our analyses. We did not ask about any potentially illegal behaviors (such as vacation cutting or FGM occurring in the US) due to child maltreatment laws and mandated reporting requirements. Finally, although interviewers were trained to assist women with navigating the WHNS Resource Website, some women may have been unable to access the website due to digital and/or computer literacy limitations. We do not know if there are differences between the participants who did and did not access the WHNS Resource Website.

## Conclusion

WHNS advances research and fills important gaps in the literature related to the health and FGM characteristics of U.S. women aged 18–49 who were born, or whose mothers were born in a country where FGM is prevalent. The VBS/RDS approach enabled the recruitment of a diverse sample. The WHNS accomplished the study goal of implementing a survey among women living in the United States, from countries where FGM is prevalent. Future studies may consider using a similar VBS/RDS approach with community engagement from study design through dissemination activities. Findings from future analyses may potentially inform programmatic activities in the study communities. The WHNS findings have been disseminated to communities who helped implement the study (some of which include social services and health workers providing services within the communities). Each community partner will decide if and how they will use findings from the study to support their ongoing activities. These activities include: doula workshops, community conversations / listening sessions, creating and translating infographics, workshops, webinars, and presentations.

Finally, future analysis of WHNS data will include analyzing the reproductive health related variables. This analysis, may contribute to the formulation of evidence-based reproductive health strategies that address the public health needs of U.S. women from countries where FGM is prevalent especially women who may have experienced or been at risk for FGM.

## Supporting information

S1 Table Supplemental material WHNS seed and sprout information. (DOCX)

S1 File Supplemental material WHNS screener. (PDF)

S2 File Supplemental material WHNS questionnaire. (PDF)

S3 File Supplemental material WHNS FGM type. (DOCX)
