# Supplementary material for: The women’s health needs study among women from countries with high prevalence of female genital mutilation living in the United States: Design, methods, and participant characteristics
Source: PLoS One. 2024 May 31;19(5):e0302820. doi: 10.1371/journal.pone.0302820 (PMC11142442; doi:10.1371/journal.pone.0302820)
Supplement: S1 Table — (DOCX) [file pone.0302820.s001.docx]

S3 Table Supplemental Material WHNS Seed and Sprout Information

| **Women Recruited** | | | | | |  |  |  |  |  |  |  |  |  |  |  |
| --- | --- | --- | --- | --- | --- | --- | --- | --- | --- | --- | --- | --- | --- | --- | --- | --- |
| Seed* | | | | 35% | |  |  |  |  |  |  |  |  |  |  |  |
| Sprout** | | | | 65% | |  |  |  |  |  |  |  |  |  |  |  |
| **Completed Interviews** | | | | | | |  |  |  |  |  |  |  |  |  |  |
| Seed* | | 34% | | | | |  |  |  |  |  |  |  |  |  |  |
| Sprout** | | 66% | | | | |  |  |  |  |  |  |  |  |  |  |
| **Seed Sprout and Age** | | | | | | | | | | | | |  |  |  |  |
|  | | | Age Categories | | | | | | | | | |  |  |  |  |
|  |  |  | 18-24 | | 25-29 | | | 30-34 | 35-39 | | | 40-49 |  |  |  |  |
| Seed* | | | 18.99% | | 12.15% | | | 15.70% | 17.97% | | | 35.19% |  |  |  |  |
| Sprout** | | | 24.83% | | 13.03% | | | 15.33% | 23.20% | | | 23.61% |  |  |  |  |
| **Seed Sprout and Education** | | | | | | | | | | | | | | | | |
|  | What is the highest level of schooling you have completed? | | | | | | | | | | | | | | | |
|  | No formal school | | | | Less than a high school diploma | | | High school diploma | | Some college | Associate’s degree | | | Bachelor’s degree | Don’t Know | Prefer not to Answer |
| Seed* | 9.62 | | | | 17.72 | | | 22.78 | | 18.99 | 7.34 | | | 23.29 | 0.25 | 0 |
| Sprout** | 5.29 | | | | 17.91 | | | 29.04 | | 19.67 | 9.91 | | | 18.05 | 0 | 0.14 |

| *Women recruited to the study from one of the study sites (VBS)  **Women referred to the study by a participant (RDS) |
| --- |
