# Supplementary material for: The women’s health needs study among women from countries with high prevalence of female genital mutilation living in the United States: Design, methods, and participant characteristics
Source: PLoS One. 2024 May 31;19(5):e0302820. doi: 10.1371/journal.pone.0302820 (PMC11142442; doi:10.1371/journal.pone.0302820)
Supplement: S1 File — (PDF) [file pone.0302820.s002.pdf]

Form Approved
OMB Number: 0920-1264
Expiration Date: 05/31/2022

Women's Health Needs STUDY

Questionnaire: English

Public reporting burden of this collection of information is estimated to average 45 minute per response, including the time for reviewing instructions, searching existing data sources, gathering and maintaining the data needed, and completing and reviewing the collection of information. An agency may not conduct or sponsor, and a person is not required to respond to a collection of information unless it displays a currently valid OMB control number. Send comments regarding this burden estimate or any other aspect of this collection of information, including suggestions for reducing this burden to CDC/ATSDR Reports Clearance Officer; 1600 Clifton Road NE, MS D-74, Atlanta, Georgia 30333; ATTN: PRA (0920-1264).

SECTION A: SCREENER

1. Have you previously participated in the Women's Health Needs Study (WHNS)?

1 ☐ **Yes** *[END SURVEY]*
2 ☐ **No**

2. Which of the following languages do you speak?

1 ☐ **Amharic**
2 ☐ **Arabic**
3 ☐ **English**
4 ☐ **Somali**
5 ☐ **Swahili**
6 ☐ **Other,** *please specify:*

3. Which language would you prefer to use for this interview? *[INTERVIEWER NOTE: IF YOU DO NOT SPEAK THE LANGUAGE PREFERRED, MAKE NOTE AND END INTERVIEW]*.

4. In which country were you born?

1 ☐ Don't Know
2 ☐ Prefer not to answer

[SKIP LOGIC: IF RESPONDENT MENTIONS EGYPT, ERITREA, KENYA, SOMALIA, or ETHIOPIA,,GUINEA, LIBERIA, NIGERIA, SIERRA LEONE or SUDAN. GO TO Q6]

5. Have you ever lived in any of the following countries?

Egypt	1 ☐ **Yes**	2 ☐ **No**	3 ☐ Don't Know	4 ☐ Prefer not to answer
Eritrea	1 ☐ **Yes**	2 ☐ **No**	3 ☐ Don't Know	4 ☐ Prefer not to answer
Kenya	1 ☐ **Yes**	2 ☐ **No**	3 ☐ Don't Know	4 ☐ Prefer not to answer
Somalia	1 ☐ **Yes**	2 ☐ **No**	3 ☐ Don't Know	4 ☐ Prefer not to answer
Ethiopia	1 ☐ **Yes**	2 ☐ **No**	3 ☐ Don't Know	4 ☐ Prefer not to answer
Guinea	1 ☐ **Yes**	2 ☐ **No**	3 ☐ Don't Know	4 ☐ Prefer not to answer
Liberia	1 ☐ **Yes**	2 ☐ **No**	3 ☐ Don't Know	4 ☐ Prefer not to answer
Nigeria	1 ☐ **Yes**	2 ☐ **No**	3 ☐ Don't Know	4 ☐ Prefer not to answer
Siera Leone	1 ☐ **Yes**	2 ☐ **No**	3 ☐ Don't Know	4 ☐ Prefer not to answer
Sudan	1 ☐ **Yes**	2 ☐ **No**	3 ☐ Don't Know	4 ☐ Prefer not to answer

6. In which country was your <u>father</u> born?

1 ☐ Don't Know
2 ☐ Prefer not to answer

7. In which country was your <u>mother</u> born?

1 ☐ Don't Know
2 ☐ Prefer not to answer

[SKIP LOGIC: IF RESPONDENT MENTIONS EGYPT, ERITREA, KENYA, SOMALIA, or or ETHIOPIA,,GUINEA, LIBERIA, NIGERIA, SIERRA LEONE or SUDAN. \ GO TO Q9]

8. Has your mother lived in any of the following countries?

Egypt	1 ☐ **Yes**	2 ☐ **No**	3 ☐ Don't Know	4 ☐ Prefer not to answer
Eritrea	1 ☐ **Yes**	2 ☐ **No**	3 ☐ Don't Know	4 ☐ Prefer not to answer
Kenya	1 ☐ **Yes**	2 ☐ **No**	3 ☐ Don't Know	4 ☐ Prefer not to answer
Somalia	1 ☐ **Yes**	2 ☐ **No**	3 ☐ Don't Know	4 ☐ Prefer not to answer
Ethiopia	1 ☐ **Yes**	2 ☐ **No**	3 ☐ Don't Know	4 ☐ Prefer not to answer
Guinea	1 ☐ **Yes**	2 ☐ **No**	3 ☐ Don't Know	4 ☐ Prefer not to answer
Liberia	1 ☐ **Yes**	2 ☐ **No**	3 ☐ Don't Know	4 ☐ Prefer not to answer
Nigeria	1 ☐ **Yes**	2 ☐ **No**	3 ☐ Don't Know	4 ☐ Prefer not to answer
Siera Leone	1 ☐ **Yes**	2 ☐ **No**	3 ☐ Don't Know	4 ☐ Prefer not to answer
Sudan	1 ☐ **Yes**	2 ☐ **No**	3 ☐ Don't Know	4 ☐ Prefer not to answer

9. How old are you?

1 ☐ **Under 18 years**
2 ☐ **18-24**
3 ☐ **25-29**
4 ☐ **30-34**
5 ☐ **35-39**
6 ☐ **40-49**
7 ☐ **Over 49 years**
8 ☐ Prefer not to answer

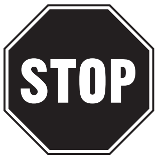

Eligible: if between 18-49 years old, birth location or country lived if respondent or mother fits criteria from country list, and translation options are necessary and available, respondent is eligible. Review consent material and proceed to full interview if consent obtained.
