# Supplementary material for: The women’s health needs study among women from countries with high prevalence of female genital mutilation living in the United States: Design, methods, and participant characteristics
Source: PLoS One. 2024 May 31;19(5):e0302820. doi: 10.1371/journal.pone.0302820 (PMC11142442; doi:10.1371/journal.pone.0302820)
Supplement: S2 File — (PDF) [file pone.0302820.s003.pdf]

Form Approved
OMB Number: 0920-1264
Expiration Date: 05/31/2022

Women's Health Needs STUDY

Questionnaire: English

Public reporting burden of this collection of information is estimated to average 45 minute per response, including the time for reviewing instructions, searching existing data sources, gathering and maintaining the data needed, and completing and reviewing the collection of information. An agency may not conduct or sponsor, and a person is not required to respond to a collection of information unless it displays a currently valid OMB control number. Send comments regarding this burden estimate or any other aspect of this collection of information, including suggestions for reducing this burden to CDC/ATSDR Reports Clearance Officer; 1600 Clifton Road NE, MS D-74, Atlanta, Georgia 30333; ATTN: PRA (0920-1264).

Interview Start Time: [ ] Hour [ ] Minute

SECTION B: BACKGROUND CHARACTERISTICS

Now we can begin. I am going to start by asking you some more questions about your background. Your answers will not be shared with anyone outside of the research team.

1. What language do you speak most often at home?

[ ]

2. What language(s) do you speak most often with your closest friends? *[INTERVIEWER NOTE: Allow for two languages to be given]*

[ ]

[ ]

3. In what country does your mother live now?

[ ]

1 ☐ Mother passed away *[GO TO Q5]*
77 ☐ Don't Know *[GO TO Q5]*
99 ☐ Prefer not to answer *[GO TO Q5]*

4. How often do you speak with your mother?

1 ☐ **Daily**
2 ☐ **2-3 times a week**
3 ☐ **Once a week**
4 ☐ **Once/twice a month**
5 ☐ **Less than once a month**
6 ☐ **Never**
77 ☐ Don't Know
99 ☐ Prefer not to answer

5. How many times have you traveled to each of the following countries? *[ENTER 0 IF RESPONDENT HAS NEVER TRAVELED TO COUNTRY].*

[ ] **Burkina Faso**
[ ] **Egypt**
[ ] **Eritrea**
[ ] **Ethiopia**
[ ] **Gambia**
[ ] **Guinea**
[ ] **Mali**
[ ] **Mauritania**
[ ] **Sierra Leone**
[ ] **Somalia**
[ ] **Sudan**

77 ☐ Don't Know
99 ☐ Prefer not to answer

6. How long ago did you move to the United States? *[INTERVIEWER NOTE: Select best option based on answer for the most recent time]*

1 ☐ **Within the last year**
2 ☐ **1-5 years ago**
3 ☐ **6-10 years ago**
4 ☐ **Over 10 years ago**
5 ☐ **Born in the U.S.** *[GO TO SECTION C]*
77 ☐ Don't Know
99 ☐ Prefer not to answer

7. How old were you when you moved to the United States?

1 ☐ **0-6 years old**
2 ☐ **7-12 years old**
3 ☐ **13-17 years old**
4 ☐ **18 years or older**
77 ☐ Don't Know
99 ☐ Prefer not to answer

SECTION C: MARRIAGE AND HOUSEHOLD

Next, I am going to ask you questions about your marital status and living arrangements.

8. Including yourself, how many people live in your household now? *Please count children and elders. <u>Do NOT count</u> people staying in the home for less than one month.*

[ ][ ]

77 ☐ Don't Know
99 ☐ Prefer not to answer

9. Which of the following describes your current marital status? *Are you married, living with a partner, widowed, divorced, separated, or have you never been married?*

1 ☐ **Married**
2 ☐ **Widowed**
3 ☐ **Divorced**
4 ☐ **Separated**
5 ☐ **Not married, but living with a partner**
6 ☐ **Never married/lived with partner** *[GO TO Q14]*
99 ☐ Prefer not to answer *[GO TO Q14]*

10. How old were you when you first got married or started living with a partner?

1 ☐ **Under 18 years**
2 ☐ **18-24 years**
3 ☐ **25-29 years**
4 ☐ **30-39 years**
5 ☐ **40-49 years**
6 ☐ **Over 49 years**
77 ☐ Don't Know
99 ☐ Prefer not to answer

11. How old was your husband/partner when you first got married or started living together?

1 ☐ **Under 18 years**
2 ☐ **18-24 years**
3 ☐ **25-29 years**
4 ☐ **30-39 years**
5 ☐ **40-49 years**
6 ☐ **Over 49 years**
77 ☐ Don't Know
99 ☐ Prefer not to answer

12. In what country did your first marriage/partnership take place?

[ ]

77 ☐ Don't Know
99 ☐ Prefer not to answer

13. In what country was your husband/partner born?

[ ]

77 ☐ Don't Know
99 ☐ Prefer not to answer

SECTION D: COMMUNITY ACTIVITIES

I am now going to ask you some questions about your participation in community activities such as neighborhood organizations or groups.

14. Are you a member of any club, association, or religious organization for people from your family's home country or ethnic/cultural background?

1 ☐ **Yes**
2 ☐ **No**
3 ☐ Not sure
99 ☐ Prefer not to answer

15. When you invite people to your home, are they...

1 ☐ **Mostly people <u>from</u> my home country or ethnic/cultural background**
2 ☐ **Mostly people <u>NOT from</u> my home country or ethnic/cultural background**
3 ☐ **A mix of people <u>from AND not from</u> my home country or ethnic/cultural background**
4 ☐ **I never invite people to my home**
99 ☐ Prefer not to answer

16. Have you done any work outside of the home for pay in the <u>past 30 days</u>?

1 ☐ **Yes**
2 ☐ **No**
77 ☐ Don't Know
99 ☐ Prefer not to answer

SECTION E: HEALTH-SEEKING BEHAVIOR AND PROVIDER EXPERIENCE

Now I am going to ask you some questions about your overall health and experiences with health care, services, and providers.

17. In general, how would you describe your health? *Is it excellent, very good, good, fair, or poor?*

1 ☐ **Excellent**
2 ☐ **Very good**
3 ☐ **Good**
4 ☐ **Fair**
5 ☐ **Poor**
6 ☐ Not sure
99 ☐ Prefer not to answer

18. How many times have you gone to a clinic or hospital for health care for yourself in the past 12 months?

1 ☐ **Not at all**
2 ☐ **Once**
3 ☐ **Twice**
4 ☐ **3-5 times**
5 ☐ **More than 5 times**
77 ☐ Don't Know
99 ☐ Prefer not to answer

19. When visiting your healthcare provider, would you like to have someone present to interpret?

1 ☐ **Yes**
2 ☐ **No** *[GO TO Q22]*
3 ☐ Do not have a healthcare provider *[GO TO Q22]*
77 ☐ Don't Know *[GO TO Q22]*
99 ☐ Prefer not to answer *[GO TO Q22]*

20. During your last visit, was an interpreter offered to you?

1 ☐ **Yes**
2 ☐ **No**
77 ☐ Don't Know
99 ☐ Prefer not to answer

21. Who usually serves as an interpreter for you?

1 ☐ **My health provider**
2 ☐ **Professional interpreter**
3 ☐ **A staff person**
4 ☐ **A female friend or relative**
5 ☐ **My husband/partner, or other male relative**
6 ☐ Other, *please specify:*

99 ☐ Prefer not to answer

22. Are you currently covered by any of the following types of health insurance?

1 ☐ **A plan purchased through an employer or union (includes plans purchased through another person's employer)**
2 ☐ **A plan that you or a family member buys on their own**
3 ☐ **Medicaid or other state or federal program**
4 ☐ **Some other source,** *please specify:*

5 ☐ **I do not currently have health insurance**
77 ☐ Don't Know
99 ☐ Prefer not to answer

23. During the past 12 months, was there any time when you needed medical care but didn't get it because you couldn't afford it?

1 ☐ **Yes**
2 ☐ **No**
77 ☐ Don't Know
99 ☐ Prefer not to answer

SECTION F: WOMEN'S HEALTH AND PREGNANCY OUTCOMES

I am now going to ask you questions about family planning and your sexual health.

24. Have you ever used any contraceptives or birth control methods to avoid or delay getting pregnant?

1 ☐ **Yes**
2 ☐ **No** *[GO TO Q26]*
77 ☐ Don't Know *[GO TO Q26]*
99 ☐ Prefer not to answer *[GO TO Q26]*

25. Which method(s) have you <u>ever</u> used? Have you used this method in the past 30 days?

	Ever Used?	Used in past 30 days?
**a.** Female sterilization (tubes tied)	1 ☐ Yes<br>2 ☐ No	
**b.** Male sterilization	1 ☐ Yes<br>2 ☐ No	
**c.** Contraceptive implant (Nexplanon, Jadelle, Sino, Implant, Implanon)	1 ☐ Yes →<br>2 ☐ No	1 ☐ Yes<br>2 ☐ No
**d.** IUD (for example, Paragard, Mirena, Skyla, Liletta)	1 ☐ Yes →<br>2 ☐ No	1 ☐ Yes<br>2 ☐ No
**e.** Shots/Injections (for example, Depo-Provera)	1 ☐ Yes →<br>2 ☐ No	1 ☐ Yes<br>2 ☐ No
**f.** Birth control pills (daily pills, any kind)	1 ☐ Yes →<br>2 ☐ No	1 ☐ Yes<br>2 ☐ No
**g.** Contraceptive patch (Ortho Evra, Xulane)	1 ☐ Yes →<br>2 ☐ No	1 ☐ Yes<br>2 ☐ No
**h.** Contraceptive ring (NuvaRing)	1 ☐ Yes →<br>2 ☐ No	1 ☐ Yes<br>2 ☐ No
**i.** Male condoms	1 ☐ Yes →<br>2 ☐ No	1 ☐ Yes<br>2 ☐ No
**j.** Diaphragm	1 ☐ Yes →<br>2 ☐ No	1 ☐ Yes<br>2 ☐ No
**k.** Female condoms	1 ☐ Yes →<br>2 ☐ No	1 ☐ Yes<br>2 ☐ No
**l.** Foam, jelly, or cream	1 ☐ Yes →<br>2 ☐ No	1 ☐ Yes<br>2 ☐ No
**m.** Emergency contraception (morning after pill)	1 ☐ Yes →<br>2 ☐ No	1 ☐ Yes<br>2 ☐ No
**n.** Not having sex at certain times (rhythm or natural family planning)	1 ☐ Yes →<br>2 ☐ No	1 ☐ Yes<br>2 ☐ No
**o.** Withdrawal (pulling out)	1 ☐ Yes →<br>2 ☐ No	1 ☐ Yes<br>2 ☐ No
**p.** Other, *please specify:* ________	1 ☐ Yes →<br>2 ☐ No	1 ☐ Yes<br>2 ☐ No

26. In the past 12 months, have you had trouble getting a contraceptive or birth control method you wanted?

- 1 ☐ **Yes**
- 2 ☐ **No**
- 3 ☐ **I did not want a birth control method**
- 77 ☐ Don't Know
- 99 ☐ Prefer not to answer

27. When was your last pelvic exam and/or pap smear?

- 1 ☐ **Within past year**
- 2 ☐ **1-3 years ago**
- 3 ☐ **4 to 5 years ago**
- 4 ☐ **More than 5 years ago**
- 5 ☐ **Never**
- 77 ☐ Don't Know
- 99 ☐ Prefer not to answer

28. How old were you when you had sexual intercourse for the first time? *[READ IF NECESSARY: Do not count oral sex, anal sex, heavy petting, or other forms of sexual activity that do not involve vaginal penetration. Do not count sex with a female partner].*

- 1 ☐ **Under 18 years**
- 2 ☐ **18-24**
- 3 ☐ **25-29 years**
- 4 ☐ **30-39 years**
- 5 ☐ **40-49 years**
- 6 ☐ **Over 49 years**
- 7 ☐ **Never had sexual intercourse** *[GO TO Q37]*
- 77 ☐ Don't Know
- 99 ☐ Prefer not to answer

SECTION G: WOMEN'S HEALTH AND PREGNANCY OUTCOMES

To finish up our questions about health and health care, we have a few questions for you about pregnancy and prenatal care. *Prenatal care is when you get checkups from a doctor, nurse, or midwife while you are pregnant.*

29. Are you pregnant now?

- 1 ☐ **Yes**
- 2 ☐ **No** *[GO TO Q31]*
- 77 ☐ Don't Know *[GO TO Q31]*
- 99 ☐ Prefer not to answer *[GO TO Q31]*

30. Have you had prenatal care for this pregnancy?

- 1 ☐ **Yes**
- 2 ☐ **No**
- 99 ☐ Prefer not to answer

Now we have some questions about your children.

31. How many children have you given birth to that were born alive?

☐☐ *[IF 0, GO TO Q37]*

- 77 ☐ Don't Know *[GO TO Q37]*
- 99 ☐ Prefer not to answer *[GO TO Q37]*

Now I will ask a few questions about each child you had beginning with the oldest one.

	32. In what month and year was this child born?	33. Is this child still alive?	34. Was this child born in the U.S.?	35. How many weeks (or months) pregnant were you at the time of your first prenatal care visit?	36. Was this baby delivered by caesarean section (c-section)?
Child 1	Month: ☐☐<br>Year: ☐☐☐☐<br>99 ☐ Prefer not to answer	1 ☐ Yes<br>2 ☐ No<br>99 ☐ Prefer not to answer	1 ☐ Yes<br>2 ☐ No<br>99 ☐ Prefer not to answer	☐☐<br>1 ☐ Weeks<br>2 ☐ Months<br>3 ☐ No Prenatal care<br>77 ☐ Don't Know<br>99 ☐ Prefer not to answer	1 ☐ Yes<br>2 ☐ No<br>99 ☐ Prefer not to answer<br>*[GO TO NEXT CHILD OR TO Q37 IF NO MORE CHILDREN]*
Child 2	Month: ☐☐<br>Year: ☐☐☐☐<br>99 ☐ Prefer not to answer	1 ☐ Yes<br>2 ☐ No<br>99 ☐ Prefer not to answer	1 ☐ Yes<br>2 ☐ No<br>99 ☐ Prefer not to answer	☐☐<br>1 ☐ Weeks<br>2 ☐ Months<br>3 ☐ No Prenatal care<br>77 ☐ Don't Know<br>99 ☐ Prefer not to answer	1 ☐ Yes<br>2 ☐ No<br>99 ☐ Prefer not to answer<br>*[GO TO NEXT CHILD OR TO Q37 IF NO MORE CHILDREN]*
Child 3	Month: ☐☐<br>Year: ☐☐☐☐<br>99 ☐ Prefer not to answer	1 ☐ Yes<br>2 ☐ No<br>99 ☐ Prefer not to answer	1 ☐ Yes<br>2 ☐ No<br>99 ☐ Prefer not to answer	☐☐<br>1 ☐ Weeks<br>2 ☐ Months<br>3 ☐ No Prenatal care<br>77 ☐ Don't Know<br>99 ☐ Prefer not to answer	1 ☐ Yes<br>2 ☐ No<br>99 ☐ Prefer not to answer<br>*[GO TO NEXT CHILD OR TO Q37 IF NO MORE CHILDREN]*
Child 4	Month: ☐☐<br>Year: ☐☐☐☐<br>99 ☐ Prefer not to answer	1 ☐ Yes<br>2 ☐ No<br>99 ☐ Prefer not to answer	1 ☐ Yes<br>2 ☐ No<br>99 ☐ Prefer not to answer	☐☐<br>1 ☐ Weeks<br>2 ☐ Months<br>3 ☐ No Prenatal care<br>77 ☐ Don't Know<br>99 ☐ Prefer not to answer	1 ☐ Yes<br>2 ☐ No<br>99 ☐ Prefer not to answer<br>*[GO TO NEXT CHILD OR TO Q37 IF NO MORE CHILDREN]*
Child 5	Month: ☐☐<br>Year: ☐☐☐☐<br>99 ☐ Prefer not to answer	1 ☐ Yes<br>2 ☐ No<br>99 ☐ Prefer not to answer	1 ☐ Yes<br>2 ☐ No<br>99 ☐ Prefer not to answer	☐☐<br>1 ☐ Weeks<br>2 ☐ Months<br>3 ☐ No Prenatal care<br>77 ☐ Don't Know<br>99 ☐ Prefer not to answer	1 ☐ Yes<br>2 ☐ No<br>99 ☐ Prefer not to answer<br>*[GO TO NEXT CHILD OR TO Q37 IF NO MORE CHILDREN]*
Child 6	Month: ☐☐<br>Year: ☐☐☐☐<br>99 ☐ Prefer not to answer	1 ☐ Yes<br>2 ☐ No<br>99 ☐ Prefer not to answer	1 ☐ Yes<br>2 ☐ No<br>99 ☐ Prefer not to answer	☐☐<br>1 ☐ Weeks<br>2 ☐ Months<br>3 ☐ No Prenatal care<br>77 ☐ Don't Know<br>99 ☐ Prefer not to answer	1 ☐ Yes<br>2 ☐ No<br>99 ☐ Prefer not to answer<br>*[GO TO NEXT CHILD OR TO Q37 IF NO MORE CHILDREN]*
Child 7	Month: ☐☐<br>Year: ☐☐☐☐<br>99 ☐ Prefer not to answer	1 ☐ Yes<br>2 ☐ No<br>99 ☐ Prefer not to answer	1 ☐ Yes<br>2 ☐ No<br>99 ☐ Prefer not to answer	☐☐<br>1 ☐ Weeks<br>2 ☐ Months<br>3 ☐ No Prenatal care<br>77 ☐ Don't Know<br>99 ☐ Prefer not to answer	1 ☐ Yes<br>2 ☐ No<br>99 ☐ Prefer not to answer<br>*[GO TO NEXT CHILD OR TO Q37 IF NO MORE CHILDREN]*

**Child 8**	Month:<br>Year:<br>99 ☐ Prefer not to answer	1 ☐ **Yes**<br>2 ☐ **No**<br>99 ☐ Prefer not to answer	1 ☐ **Yes**<br>2 ☐ **No**<br>99 ☐ Prefer not to answer		1 ☐ **Weeks**<br>2 ☐ **Months**<br>3 ☐ **No Prenatal care**<br>77 ☐ Don't Know<br>99 ☐ Prefer not to answer	1 ☐ **Yes**<br>2 ☐ **No**<br>99 ☐ Prefer not to answer<br>*[GO TO NEXT CHILD OR TO Q37 IF NO MORE CHILDREN]*
**Child 9**	Month:<br>Year:<br>99 ☐ Prefer not to answer	1 ☐ **Yes**<br>2 ☐ **No**<br>99 ☐ Prefer not to answer	1 ☐ **Yes**<br>2 ☐ **No**<br>99 ☐ Prefer not to answer		1 ☐ **Weeks**<br>2 ☐ **Months**<br>3 ☐ **No Prenatal care**<br>77 ☐ Don't Know<br>99 ☐ Prefer not to answer	1 ☐ **Yes**<br>2 ☐ **No**<br>99 ☐ Prefer not to answer<br>*[GO TO NEXT CHILD OR TO Q37 IF NO MORE CHILDREN]*
**Child 10**	Month:<br>Year:<br>99 ☐ Prefer not to answer	1 ☐ **Yes**<br>2 ☐ **No**<br>99 ☐ Prefer not to answer	1 ☐ **Yes**<br>2 ☐ **No**<br>99 ☐ Prefer not to answer		1 ☐ **Weeks**<br>2 ☐ **Months**<br>3 ☐ **No Prenatal care**<br>77 ☐ Don't Know<br>99 ☐ Prefer not to answer	1 ☐ **Yes**<br>2 ☐ **No**<br>99 ☐ Prefer not to answer<br>*[GO TO NEXT CHILD OR TO Q37 IF NO MORE CHILDREN]*
**Child 11**	Month:<br>Year:<br>99 ☐ Prefer not to answer	1 ☐ **Yes**<br>2 ☐ **No**<br>99 ☐ Prefer not to answer	1 ☐ **Yes**<br>2 ☐ **No**<br>99 ☐ Prefer not to answer		1 ☐ **Weeks**<br>2 ☐ **Months**<br>3 ☐ **No Prenatal care**<br>77 ☐ Don't Know<br>99 ☐ Prefer not to answer	1 ☐ **Yes**<br>2 ☐ **No**<br>99 ☐ Prefer not to answer<br>*[GO TO NEXT CHILD OR TO Q37 IF NO MORE CHILDREN]*
**Child 12**	Month:<br>Year:<br>99 ☐ Prefer not to answer	1 ☐ **Yes**<br>2 ☐ **No**<br>99 ☐ Prefer not to answer	1 ☐ **Yes**<br>2 ☐ **No**<br>99 ☐ Prefer not to answer		1 ☐ **Weeks**<br>2 ☐ **Months**<br>3 ☐ **No Prenatal care**<br>77 ☐ Don't Know<br>99 ☐ Prefer not to answer	1 ☐ **Yes**<br>2 ☐ **No**<br>99 ☐ Prefer not to answer<br>*[GO TO NEXT CHILD OR TO Q37 IF NO MORE CHILDREN]*
**Child 13**	Month:<br>Year:<br>99 ☐ Prefer not to answer	1 ☐ **Yes**<br>2 ☐ **No**<br>99 ☐ Prefer not to answer	1 ☐ **Yes**<br>2 ☐ **No**<br>99 ☐ Prefer not to answer		1 ☐ **Weeks**<br>2 ☐ **Months**<br>3 ☐ **No Prenatal care**<br>77 ☐ Don't Know<br>99 ☐ Prefer not to answer	1 ☐ **Yes**<br>2 ☐ **No**<br>99 ☐ Prefer not to answer<br>*[GO TO NEXT CHILD OR TO Q37 IF NO MORE CHILDREN]*
**Child 14**	Month:<br>Year:<br>99 ☐ Prefer not to answer	1 ☐ **Yes**<br>2 ☐ **No**<br>99 ☐ Prefer not to answer	1 ☐ **Yes**<br>2 ☐ **No**<br>99 ☐ Prefer not to answer		1 ☐ **Weeks**<br>2 ☐ **Months**<br>3 ☐ **No Prenatal care**<br>77 ☐ Don't Know<br>99 ☐ Prefer not to answer	1 ☐ **Yes**<br>2 ☐ **No**<br>99 ☐ Prefer not to answer<br>*[GO TO NEXT CHILD OR TO Q37 IF NO MORE CHILDREN]*
**Child 15**	Month:<br>Year:<br>99 ☐ Prefer not to answer	1 ☐ **Yes**<br>2 ☐ **No**<br>99 ☐ Prefer not to answer	1 ☐ **Yes**<br>2 ☐ **No**<br>99 ☐ Prefer not to answer		1 ☐ **Weeks**<br>2 ☐ **Months**<br>3 ☐ **No Prenatal care**<br>77 ☐ Don't Know<br>99 ☐ Prefer not to answer	1 ☐ **Yes**<br>2 ☐ **No**<br>99 ☐ Prefer not to answer<br>*[GO TO NEXT CHILD OR TO Q37 IF NO MORE CHILDREN]*

SECTION H: FGM/C

In a number of countries, there is a practice called circumcision in which a girl or young woman may have part of her genitals cut. Now I would like to ask you some questions about your knowledge and experiences with female circumcision.

37. Do you come from a family that has practiced the tradition of female circumcision?

- 1 ☐ **Yes**
- 2 ☐ **No**
- 77 ☐ Don't Know
- 99 ☐ Prefer not to answer

38. Does your husband/partner come from a family that has practiced the tradition of female circumcision?

- 1 ☐ **Yes**
- 2 ☐ **No**
- 3 ☐ Do not have husband/partner
- 77 ☐ Don't Know
- 99 ☐ Prefer not to answer

39. Have you ever been circumcised?

- 1 ☐ **Yes**
- 2 ☐ **No** *[GO TO Q50]*
- 77 ☐ Don't Know *[GO TO Q50]*
- 99 ☐ Prefer not to answer *[GO TO Q50]*

40. How old were you when first circumcised?

- 1 ☐ **Less than 1 year old**
- 2 ☐ **1-4 years old**
- 3 ☐ **5-9 years old**
- 4 ☐ **10-14 years old**
- 5 ☐ **15-19 years old**
- 6 ☐ **More than 19 years old**
- 7 ☐ Too young to remember
- 77 ☐ Don't Know
- 99 ☐ Prefer not to answer

41. Now I would like to ask you some more questions about your circumcision. Was any flesh removed from the genital area?

- 1 ☐ **Yes** *[GO TO Q43]*
- 2 ☐ **No**
- 77 ☐ Don't Know
- 99 ☐ Prefer not to answer

42. Was the genital area nicked without removing any flesh?

- 1 ☐ **Yes**
- 2 ☐ **No**
- 77 ☐ Don't Know
- 99 ☐ Prefer not to answer

43. Was your genital area sewn closed?

- 1 ☐ **Yes**
- 2 ☐ **No**
- 77 ☐ Don't Know
- 99 ☐ Prefer not to answer

44. What kind of circumcision do you have?

- 1 ☐ **Type 1**
- 2 ☐ **Type 2**
- 3 ☐ **Type 3**
- 4 ☐ Other
- 77 ☐ Don't Know
- 99 ☐ Prefer not to answer

45. Have you ever had any problems related to your circumcision?

- 1 ☐ **Yes**
- 2 ☐ **No** *[GO TO Q47]*
- 77 ☐ Don't Know *[GO TO Q47]*
- 99 ☐ Prefer not to answer *[GO TO Q47]*

46. Please describe what problems occurred.

[INTERVIEWER NOTE: DO NOT READ RESPONSES OUT LOUD. SELECT ALL OPTIONS RESPONDENT MENTIONS OR SELECT OTHER AND WRITE IN OPEN ENDED BOX].

- 1 ☐ Difficulty passing menstrual blood
- 2 ☐ Difficulty passing urine
- 3 ☐ Pain with urination
- 4 ☐ Recurrent Urinary Tract Infections
- 5 ☐ Pain with sex
- 6 ☐ Bleeding with sex
- 7 ☐ Emergency C-section
- 8 ☐ Postpartum Hemorrhage
- 9 ☐ Extensive vaginal tears from childbirth
- 10 ☐ Other, *please specify:* ______
- 77 ☐ Don't Know
- 99 ☐ Prefer not to answer

47. Would you feel comfortable talking about your circumcision with a health care provider?

- 1 ☐ **Yes**
- 2 ☐ **No**
- 77 ☐ Don't Know
- 99 ☐ Prefer not to answer

48. Have you ever talked with a health care provider about your circumcision?

- 1 ☐ **Yes**
- 2 ☐ **No** *[GO TO Q50]*
- 77 ☐ Don't Know *[GO TO Q50]*
- 99 ☐ Prefer not to answer *[GO TO Q50]*

49. Who started the conversation about your circumcision, you or the health care provider?

- 1 ☐ **You**
- 2 ☐ **The health care provider**
- 77 ☐ Don't Know
- 99 ☐ Prefer not to answer

Have you ever experienced any of these health issues or conditions?

50. Have you ever had...?

		Is this an ongoing problem?	Did you seek professional health care for this?	Were you satisfied with how the problem was addressed?
**a. Difficulty passing menstrual blood**	1 ☐ **Yes** → 2 ☐ **No** *[GO TO Q50B]* 77 ☐ Don't Know *[GO TO Q50B]*	1 ☐ **Yes** → 2 ☐ **No** → 77 ☐ Don't Know →	1 ☐ **Yes** → 2 ☐ **No** *[GO TO Q50B]* 3 ☐ Not treatable by a doctor *[GO TO Q50B]* 77 ☐ Don't Know *[GO TO Q50B]*	1 ☐ **Yes** 2 ☐ **No** 77 ☐ Don't Know
**b. Difficulty passing urine**	1 ☐ **Yes** → 2 ☐ **No** *[GO TO Q50C]* 77 ☐ Don't Know *[GO TO Q50C]*	1 ☐ **Yes** → 2 ☐ **No** → 77 ☐ Don't Know →	1 ☐ **Yes** → 2 ☐ **No** *[GO TO Q50C]* 3 ☐ Not treatable by a doctor *[GO TO Q50C]* 77 ☐ Don't Know *[GO TO Q50C]*	1 ☐ **Yes** 2 ☐ **No** 77 ☐ Don't Know
**c. Pain with urination**	1 ☐ **Yes** → 2 ☐ **No** *[GO TO Q50D]* 77 ☐ Don't Know *[GO TO Q50D]*	1 ☐ **Yes** → 2 ☐ **No** → 77 ☐ Don't Know →	1 ☐ **Yes** → 2 ☐ **No** *[GO TO Q50D]* 3 ☐ Not treatable by a doctor *[GO TO Q50D]* 77 ☐ Don't Know *[GO TO Q50D]*	1 ☐ **Yes** 2 ☐ **No** 77 ☐ Don't Know
**d. Recurrent Urinary Tract Infections**	1 ☐ **Yes** → 2 ☐ **No** *[GO TO Q51A]* 77 ☐ Don't Know *[GO TO Q51A]*	1 ☐ **Yes** → 2 ☐ **No** → 77 ☐ Don't Know →	1 ☐ **Yes** → 2 ☐ **No** *[GO TO Q51A]* 3 ☐ Not treatable by a doctor *[GO TO Q51A]* 77 ☐ Don't Know *[GO TO Q51A]*	1 ☐ **Yes** 2 ☐ **No** 77 ☐ Don't Know

51. Have you ever...?

		Is this an ongoing problem?	Did you seek professional health care for this?	Were you satisfied with how the problem was addressed?
**a. Felt sad for many weeks at a time**	1 ☐ **Yes** → 2 ☐ **No** *[GO TO Q52A]* 77 ☐ Don't Know *[GO TO Q52A]*	1 ☐ **Yes** → 2 ☐ **No** → 77 ☐ Don't Know →	1 ☐ **Yes** → 2 ☐ **No** *[GO TO Q52A]* 3 ☐ Not treatable by a doctor *[GO TO Q52A]* 77 ☐ Don't Know *[GO TO Q52A]*	1 ☐ **Yes** 2 ☐ **No** 77 ☐ Don't Know

52. Have you ever had...?

		Is this an ongoing problem?	Did you seek professional health care for this?	Were you satisfied with how the problem was addressed?
a. Pain with sex	1 ☐ Yes ⟶<br>2 ☐ No [GO TO Q52B]<br>3 ☐ Never had sex [GO TO Q53]<br>77 ☐ Don't Know [GO TO Q52B]<br>99 ☐ Prefer not to answer [GO TO Q52B]	1 ☐ Yes ⟶<br>2 ☐ No ⟶<br>77 ☐ Don't Know ⟶	1 ☐ Yes ⟶<br>2 ☐ No [GO TO Q52B]<br>3 ☐ Not treatable by a doctor [GO TO Q52B]<br>77 ☐ Don't Know [GO TO Q52B]	1 ☐ Yes<br>2 ☐ No<br>77 ☐ Don't Know
b. Bleeding with sex	1 ☐ Yes ⟶<br>2 ☐ No [GO TO Q53A]<br>77 ☐ Don't Know [GO TO Q53A]<br>99 ☐ Prefer not to answer [GO TO Q53A]	1 ☐ Yes ⟶<br>2 ☐ No ⟶<br>77 ☐ Don't Know ⟶	1 ☐ Yes ⟶<br>2 ☐ No [GO TO Q53A]<br>3 ☐ Not treatable by a doctor [GO TO Q53A]<br>77 ☐ Don't Know [GO TO Q53A]	1 ☐ Yes<br>2 ☐ No<br>77 ☐ Don't Know

53. Have you ever had a/an...?

a. Emergency C-section	1 ☐ Yes	2 ☐ No	77 ☐ Don't Know	3 ☐ Never had live birth [GO TO Q54]
b. Postpartum hemorrhage	1 ☐ Yes	2 ☐ No	77 ☐ Don't Know	
c. Extensive vaginal tears from childbirth	1 ☐ Yes	2 ☐ No	77 ☐ Don't Know	

SECTION I: FGC BELIEFS

I am now going to ask you some questions about your beliefs and opinions about female circumcision.

54. Which of the following best describes your views about female circumcision? Would you say...

1 ☐ **It should be stopped**
2 ☐ **It should continue as is**
3 ☐ **Depends on the family**
4 ☐ **I have mixed feelings about it**
5 ☐ Other, *please specify:*

77 ☐ Don't Know
99 ☐ Prefer not to answer

55. Has your opinion about female circumcision changed in any way since you moved to the U.S.?

1 ☐ **Yes**
2 ☐ **No** [GO TO Q57]
3 ☐ Not applicable, born in U.S. [GO TO Q57]
4 ☐ Not applicable, did not have opinion before moving to U.S. [GO TO Q57]
77 ☐ Don't Know [GO TO Q57]
99 ☐ Prefer not to answer [GO TO Q57]

56. How has your opinion changed? Would you say your opinion is...

1 ☐ **More accepting of female circumcision**
2 ☐ **Less accepting of female circumcision**
77 ☐ Don't Know
99 ☐ Prefer not to answer

57. Do you believe that female circumcision is required by your religion?

1 ☐ **Yes**
2 ☐ **No**
3 ☐ No Religion
77 ☐ Don't Know
99 ☐ Prefer not to answer

58. In your opinion, can female circumcision cause any health problems for women later on (for example during pregnancy and delivery)?

1 ☐ **Yes**
2 ☐ **No**
77 ☐ Don't Know
99 ☐ Prefer not to answer

59. What are your husband/partner's views about female circumcision? Do you think he would say...

1 ☐ **It should be stopped**
2 ☐ **It should continue as is**
3 ☐ **Depends on the family**
4 ☐ **He has mixed feelings about it**
5 ☐ Other, *please specify:*

6 ☐ Do not have husband/partner
77 ☐ Don't Know
99 ☐ Prefer not to answer

SECTION J: EDUCATION

60. What is the highest level of schooling you have completed?

1 ☐ **No formal school** *[END OF SURVEY]*
2 ☐ **Less than a high school diploma**
3 ☐ **High school diploma or GED**
4 ☐ **Some college credit, no degree**
5 ☐ **Associate's degree (for example: AA, AS)**
6 ☐ **Bachelor's degree or higher (for example: BA, BS, MA, MS, MD, PhD, etc)**
77 ☐ Don't Know
99 ☐ Prefer not to answer

61. Have you ever attended school in the U.S.?

1 ☐ **Yes**
2 ☐ **No** *[END OF SURVEY]*
99 ☐ Prefer not to answer

62. Are you attending school now?

1 ☐ **Yes**
2 ☐ **No**
99 ☐ Prefer not to answer

Interview End Time: ☐ Hour ☐ Minute
