# Supplementary material for: The women’s health needs study among women from countries with high prevalence of female genital mutilation living in the United States: Design, methods, and participant characteristics
Source: PLoS One. 2024 May 31;19(5):e0302820. doi: 10.1371/journal.pone.0302820 (PMC11142442; doi:10.1371/journal.pone.0302820)
Supplement: S3 File — (DOCX) [file pone.0302820.s004.docx]

S4 Supplemental Material WHNS FGM type specific question from questionnaire (S4a Question 44), percent accessed visual aids (S4b), visual aid (S4c), and the questions used for composite variable used for analysis (S4d).

S4a Question 44

What kind of circumcision do you have?

Type 1

Type 2

Type 3

Other

Don’t Know

Prefer not to answer

S4b Percent accessed visual aids*

| **Accessed FGM/C Visual Aid for Question 44 Types of Circumcision (N=581)** | |
| --- | --- |
| Yes | 32% |
| No | 68% |

* Nearly a third of participants accessed visual aid images provided for the single question specifically about type of FGM/C (Question 44 Supplemental Material Questionnaire). We did not use this single question (Question 44 Supplemental Material Questionnaire) for this analysis and do not know if the use of visual aid images improved or changed participant’s reporting on FGM/C types.

S4c WHNS FGM/C type visual aid available for participants to access^


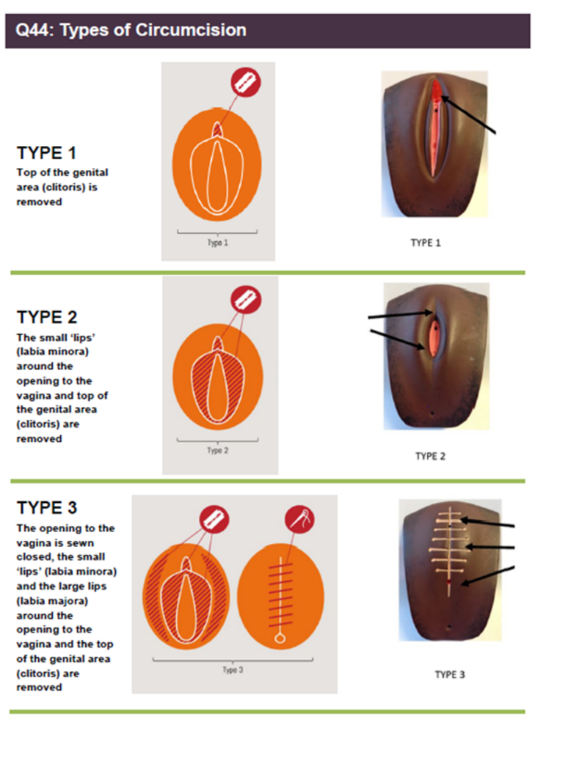


^ Visual aids developed by NORC based on original images: [Say No to Female Genital Mutilation - CodeBlue (galencentre.org)](https://codeblue.galencentre.org/2022/02/07/say-no-to-female-genital-mutilation/)

S4d Questions 41-43 FGM/C descriptive characteristics questions used to create the hierarchical categorization of self-reported FGM/C type composite variable which is used for the descriptive analysis in the paper**

Q 41: Now I would like to ask you some more questions about your circumcision.

Was any flesh removed from the genital area?

Yes *[GO TO Q43]*

No

Don’t Know

Prefer not to answer

Q42: Was the genital area nicked without removing any flesh?

Yes

No

Don’t Know

Prefer not to answer

Q43: Was your genital area sewn closed?

Yes

No

Don’t Know

Prefer not to answer

** Instead of using the single question (Question 44 Supplemental Material Questionnaire) for FGM/C type, we relied on three DHS^+^ descriptive questions (Questions 41-43 Supplemental Material Questionnaire) to create a composite variable.

^+^ICF. Demographic and Health Surveys Female Genital Cutting Module model woman’s questionnaire. 2020 [cited 2021 July 21]. Available from: <https://dhsprogram.com/pubs/pdf/DHSQM/DHS8-Module-FGCut-Qnnaire-EN-23Jan2020-DHSQM.pdf>
